# Parental Beliefs and Knowledge, Children’s Home Language Experiences, and School Readiness: The Dual Language Perspective

**DOI:** 10.3389/fpsyg.2021.661208

**Published:** 2021-05-24

**Authors:** Rufan Luo, Lulu Song, Carla Villacis, Gloria Santiago-Bonilla

**Affiliations:** ^1^Department of Psychology, Rutgers University, Camden, NJ, United States; ^2^Department of Early Childhood Education and Art Education, Brooklyn College, City University of New York, New York, NY, United States; ^3^Department of Public Policy and Administration, Rutgers University, Camden, NJ, United States

**Keywords:** dual language learners, dual language development, parental beliefs and knowledge, home dual language experiences, school readiness

## Abstract

Parental beliefs and knowledge about child development affect how they construct children’s home learning experiences, which in turn impact children’s developmental outcomes. A rapidly growing population of dual language learners (DLLs) highlights the need for a better understanding of parents’ beliefs and knowledge about dual language development and practices to support DLLs. The current study examined the dual language beliefs and knowledge of parents of Spanish-English preschool DLLs (*n* = 32). We further asked how socioeconomic and sociocultural factors were associated with parental beliefs and knowledge, and how parental beliefs and knowledge related to DLLs’ home dual language experiences and school readiness skills as rated by their teachers. Results suggested both strengths and opportunities for growth in parental beliefs and knowledge. Moreover, parents from higher-SES backgrounds reported beliefs and knowledge that were more consistent with scientific evidence. Furthermore, parental beliefs and knowledge was positively related to relative Spanish input at home and negatively related to the frequency of English language and literacy activities. However, parental beliefs and knowledge were not associated with children’s dual language output at home or the frequency of Spanish language and literacy activities. Finally, parental beliefs and knowledge were associated with children’s school readiness skills in Spanish but not in English. Together, these findings highlight the need for culturally responsive interventions and parent education programs, which must recognize both the strengths and areas of improvement in parents of DLLs and support parents to transform knowledge into high-quality language and literacy experiences that benefit DLLs.

## Introduction

In the United States, one in four preschoolers are dual language learners (DLLs), or children who are exposed to a language other than English at home (U.S. Census, 2018). Parents’ dual language practices, including their dual language use and engagement in language and literacy activities, are strong predictors of DLLs’ language and academic outcomes (Hoff et al., 2012; Song et al., 2012, 2021; McCabe et al., 2013). Parents’ practices are affected by their beliefs and knowledge (Weigel et al., 2006; Suskind et al., 2018). Furthermore, the beliefs and knowledge of DLLs’ parents are embedded in their socioeconomic and sociocultural contexts, which often present unique challenges for their practices to support DLLs (Coba-Rodriguez et al., 2020; Cycyk and Hammer, 2020). As dual language development becomes a prominent phenomenon, there is a critical need to examine how parents’ beliefs and knowledge regarding dual language development relate to DLLs’ dual language experiences at home and their developmental outcomes.

Both sociocultural perspectives (Vygotsky, 1978; Rogoff, 2003) and the bioecological theory (Bronfenbrenner and Morris, 2006) emphasize the role of contexts in shaping and affecting children’s development. According to the sociocultural theory, children develop language skills through engaging in social and language interactions with others, especially their parents (Vygotsky, 1978; Rogoff, 2003). Parents construct the social and cultural context for early learning, thereby playing a crucial role in children’s language and cognitive development (Reis et al., 2000; Tamis-LeMonda et al., 2014a). In the bioecological model (Bronfenbrenner and Morris, 2006), children’s development is embedded in a nested system of contexts. Frequent reciprocal interactions between the child and social partners (e.g., parents) in the immediate environment are proximal processes viewed as the primary mechanism for development. Furthermore, the proximal processes are affected by the characteristics of the child (e.g., age) and the environment (both immediate and more remote; e.g., socioeconomic status or SES, culture). The developmental niche theory (Super and Harkness, 1986) highlights parents’ knowledge and beliefs about child development as a modifiable, proximal factor that drives parenting practices and children’s home learning experiences. Guided by these theories, we hypothesize that parents’ beliefs and knowledge about dual language development are shaped by their socioeconomic and sociocultural backgrounds, and further affect children’s dual language experiences and developmental outcomes.

Therefore, we examined what parents of Spanish-English DLLs believe and know about dual language development and practices to support DLLs, how socioeconomic and sociocultural factors were associated with parental beliefs and knowledge, and how parental beliefs and knowledge, in turn, related to DLLs’ dual language experiences at home and school readiness skills.

### Parental Beliefs and Knowledge About Dual Language Development and Practices

There is growing knowledge regarding the characteristics of dual language development and evidence-based practices that support it (e.g., Byers-Heinlein and Lew-Williams, 2013; Hammer et al., 2014; Genesee, 2015). Research has shown that early exposure to two languages does not increase the risk for language delays or impairments (Genesee, 2015). Although DLLs may have lower levels of skills in each language compared to monolingual children (Vagh et al., 2009; Marchman et al., 2010), their knowledge (e.g., vocabulary size and grammatical skills) across the two languages is comparable to that of monolingual children (Pearson et al., 1993; Thordardottir et al., 2006; Hoff et al., 2012). Additionally, bilingual children may display behaviors unique to dual language development, such as code switching or language mixing, which do not indicate confusion (Hammer et al., 2014). Furthermore, bilingual children have been found to outperform monolingual children in certain aspects of social and cognitive development, such as theory of mind (Siegal et al., 2010) and metalinguistic awareness (Diaz and Farrar, 2018). For instance, a recent meta-analysis suggested a medium-sized bilingual advantage in children’s theory of mind ability, after adjusting for language proficiency (Schroeder, 2018).

In terms of evidence-based practices to support DLLs, parental language input in both English and the home language can be beneficial. While early English proficiency has been found to predict children’s academic growth, strong home language skills support English and academic learning, socio-emotional adjustment, and positive family relationships (Oh and Fuligni, 2010; Halle et al., 2012; Hammer et al., 2014; Tamis-LeMonda et al., 2014b). Most importantly, the quantity (e.g., number of words directed to children) and quality (e.g., back-and-forth conversations, frequent book reading activities, rich learning materials) of language environment provided by parents matter more than the type(s) of language parents use (Unsworth, 2016; Song et al., 2021). Thus, parents should speak with their children in the language(s) they are most proficient in so as to maximize the quality of their language input. In regard to school language environment, dual language programs have been found to support DLLs’ home language development without slowing down their English growth (Durán et al., 2013; Collier and Thomas, 2017; Garcia, 2018).

Yet, little is known about what parents of DLLs believe and know about dual language development and practices. Past qualitative work suggests that parents of DLLs highly value bilingualism (e.g., Farruggio, 2010; Kang, 2013; Lee et al., 2015). In particular, Latino parents consider English skills as fundamental to children’s academic success and view the use of Spanish as a bridge to English learning and a way to enhance family ties and cultural identity (Lee et al., 2015; Olivos and Lucero, 2018; Coba-Rodriguez et al., 2020; Cycyk and Hammer, 2020). Some parents of Spanish-English DLLs believe that bilingualism facilitates abstract thinking and brain development (King and Fogle, 2006). These beliefs and knowledge are in line with research that finds early skills in two languages to be associated with academic and social-emotional outcomes (Halle et al., 2012; Hammer et al., 2014; Tamis-LeMonda et al., 2014b) and certain social and cognitive advantages (Bialystok, 2009; Siegal et al., 2010).

Despite their strong desire to raise children to be bilingual, parents have uncertainty and misconceptions about how children develop dual language skills and how parents can best support them (Cycyk and Hammer, 2020). Some Latino immigrant parents believe that they are responsible for teaching children Spanish and the school should teach children English (Adair and Tobin, 2008; Lee et al., 2015), whereas others express the feeling of guilt for not providing adequate English input (Coba-Rodriguez et al., 2020). Parents of DLLs are also concerned that early exposure to two languages may confuse children, cause or worsen language delays, or hinder English acquisition and school learning (Yu, 2013; Lee et al., 2015; Olivos and Lucero, 2018; Cycyk and Hammer, 2020; Piller and Gerber, 2021), even though views of detrimental effects of bilingualism have been firmly rejected by research (Espinosa, 2013; Hammer et al., 2014). Moreover, although research has demonstrated the benefits of supporting home language at school (Bialystok, 2018), parents report mixed attitudes toward dual language programs. While some parents embrace dual language programs (Olivos and Lucero, 2018), others are worried that these programs provide insufficient English or inferior home language input (Farruggio, 2010; Lee et al., 2015).

### Parental Beliefs and Knowledge in Sociocultural Context

According to the bioecological theory of development, parents’ beliefs, knowledge, and developmental goals are heavily influenced by their ecological and sociocultural environment (Bronfenbrenner and Morris, 2006). Yet, little is known about the variations in parents’ perspectives toward dual language development. Here we asked whether parental dual language beliefs and knowledge varied by parents’ socioeconomic, immigration, and linguistic backgrounds.

Prior work suggests that parents’ native-born status as well as higher socioeconomic status (SES) and English proficiency are associated with more knowledge about infant development (Glick et al., 2009; Keels, 2009; Rowe et al., 2016) and early language and literacy development (Davis et al., 2016; Gonzalez et al., 2017; Suskind et al., 2018). This might be explained by disparities in parents’ access to professional sources of developmental knowledge (Rowe et al., 2016).

However, whether these findings apply to parents’ *dual language* beliefs and knowledge remains unclear. Qualitative work finds that parents often seek knowledge about dual language development from scientific research and professionals (King and Fogle, 2006), which might suggest an advantage for parents from higher-SES background. However, educational and health professionals sometimes provide misinformation on dual language development (Yu, 2013; Langdon, 2015). Additionally, there is some, albeit limited, evidence that parents’ personal experiences as immigrants and/or DLLs help them develop an understanding of dual language development and strategies for achieving bilingualism (King and Fogle, 2006; Lee et al., 2015). For instance, immigrant parents who recently came to the United States tended to show more positive attitudes toward heritage language maintenance than those who had lived in the country longer (Orozco, 2008; Farruggio, 2010; Barbosa, 2015). Immigrant parents who were fluent bilinguals also believed in the benefits of early dual language exposure (King and Fogle, 2006).

### Parental Beliefs and Knowledge in Relation to Children’s Home Dual Language Experiences and Developmental Outcomes

Parental beliefs and knowledge are manifested in their parenting practices and children’s home learning experiences, which contribute to children’s language and school readiness skills (Rowe et al., 2016; Suskind et al., 2018). For example, Latino mothers’ beliefs about the facilitative role of literacy activities predicted the frequency of mother-child shared-reading at home and children’s vocabulary and emergent literacy skills (Gonzalez et al., 2010; Cottone, 2012; Davis et al., 2016). However, the relation between parental dual language beliefs and knowledge and children’s dual language experiences can be more complicated. Although parents’ beliefs shape their language use strategies with DLLs (see De Houwer, 1999), multiple challenges, including parents’ limited language proficiency, the lack of resources, and children’s changing language skills and preference, can lead to discrepancies between what parents believe and what they do (Guardado, 2002; Liang, 2018).

To our knowledge, there have only been two quantitative studies examining the associations between parental dual language beliefs and knowledge and children’s dual language experiences and/or developmental outcomes. One study with Latino parents of 2- to 4-year-old children found that the more parents believed in children’s ability to learn two languages and considered teaching children Spanish and avoiding language mixing to be important, the more Spanish was used at home (Mancilla-Martinez and Lesaux, 2014). The research team also replicated this finding in parents of school-aged, Spanish-English DLLs who had limited English proficiency (Hwang et al., 2020). That is, parents who believed in children’s ability to learn two languages were more likely to construct a Spanish-dominant environment at home, presumably to compensate for the overwhelmingly English-speaking environment outside home. They also found that Spanish use at home was negatively associated with children’s conceptual vocabulary (i.e., total concepts children knew across English and Spanish). Hwang and colleagues speculated that the negative effect of parental beliefs on children’s conceptual vocabulary might be due to children’s preference to complete the conceptual vocabulary tests in English rather than Spanish, despite their limited English proficiency. Although these two studies are informative, little is known about how parental beliefs and knowledge are related to other aspects of children’s dual language experiences at home (e.g., the frequency of language/literacy activities) or child outcomes beyond vocabulary skills.

### The Current Study

Research on parental dual language beliefs and knowledge is primarily qualitative, which provides important insights into the complex and diverse perspectives of parents of DLLs. However, quantitative work can help us describe parental beliefs and knowledge at the group level and understand how parental beliefs and knowledge relate to parents’ socioeconomic and sociocultural backgrounds, children’s dual language experiences at home, and developmental outcomes. Synthesizing the themes from past work, we developed a survey to examine the beliefs and knowledge of Spanish-English DLLs’ parents and asked three research questions.

(1) What are parents’ beliefs and knowledge about dual language development and practices to support DLLs? We expected to identify both strengths and opportunities for growth in parents’ beliefs and knowledge.
(2) How do parents from different SES, immigration, and linguistic backgrounds vary in their beliefs and knowledge? Although these factors predict parents’ developmental knowledge, prior qualitative work suggests that the variations in parents’ dual language beliefs and knowledge may not follow the same pattern.
(3) How do parental beliefs and knowledge relate to the dual language use at home, the frequency of language and literacy activities in English and Spanish at home, and children’s school readiness in the two languages? We expected parental beliefs and knowledge to be associated with more Spanish use at home. Yet little is known about how parental beliefs and knowledge might relate to language and literacy activities and children’s school readiness skills.

## Methods

### Participants

Participants were 32 primary caregivers (30 mothers, 1 father, and 1 grandparent; hereafter referred to as parents) and their 3- to 5-year-old Spanish-English DLLs. All participating children were exposed to Spanish from at least one of the following social partners: mother, father, siblings, other adults in the household, and/or the child’s friends. These participants were recruited from 14 classrooms in a preschool in a metropolitan area in the United States. Seventy-five percent of the parents self-identified as Latino and 25% as African Americans. Four out of the eight African American parents were bilingual in English and Spanish. Although the other four African American parents did not speak Spanish, their children were exposed to Spanish from other people such as their father.

### Procedure

Parents were asked to fill out a questionnaire about their demographic information, dual language beliefs and knowledge, and children’s dual language experiences at home. The questionnaire was available in both English and Spanish. Children’s school readiness skills were rated by the lead teachers and teaching assistants in each classroom.

### Measurements

#### Demographic Information

Parents reported on their ethnicity, educational level, household income, native-born status, and number of years living in the United States. Parents also reported how well they could understand, speak, read, and write in English and Spanish, respectively (1-not well at all, 4-very well; adapted from the Early Childhood Longitudinal Study, Birth Cohort; see Baker, 2014). An average score was calculated to indicate parents’ proficiency in each language.

#### Parental Dual Language Beliefs and Knowledge

To assess parental beliefs and knowledge, we adapted the Questionnaire for Early Childhood Teachers of Dual Language Learners (Song et al., 2020) and developed a survey with 15 statements that were either consistent or inconsistent with the scientific evidence of dual language development and practices that support it (see Table 1). Parents rated each statement on a 4-point scale (1-strongly disagree, 4-strongly agree). The survey yielded good reliability (Cronbach’s alpha = 0.726). Ratings for statements that were inconsistent with the scientific evidence were reversely coded. A composite score was calculated for each parent by averaging their ratings for all statements, with higher values indicating beliefs and knowledge more consistent with scientific evidence. We also coded parents’ responses into 1-correct/0-incorrect and calculated the average correct rate for each item.

**TABLE 1:** Descriptive statistics for parental beliefs and knowledge about dual language development (sorted by correct rates from lowest to highest).

Statements	*M*	*SD*	Strongly disagree	Disagree	Agree	Strongly agree	Correct rate	Non-parametrics binomial test	*p*
Immigrant parents should try to speak English as much as they can so that their children will do well in United States preschools and schools.	2.62	0.79	12.5%	18.8%	62.5%	6.3%	31.3%	1.95	0.052
A parent can only support a child’s dual language development if she/he can speak English and another language (e.g., Spanish).	2.53	0.72	6.3%	40.6%	46.9%	6.3%	46.9%	0.18	0.860
English-speaking children may show academic and language delays in dual language programs.	2.42	0.81	12.9%	38.7%	41.9%	6.5%	51.6%	0.00	1.000
For dual language learners, although skills in each language may fall behind monolingual children early on, the total growth in both languages (e.g., total vocabulary of English and Spanish) is comparable to monolingual children (i.e., children who only speak English).	2.67	0.66	0.0%	43.3%	46.7%	10.0%	56.7%	0.55	0.584
Being in an English-only preschool program is the best way for a young dual language learner (a child learning two languages) to acquire English.	2.22	0.75	18.8%	40.6%	40.6%	0.0%	59.4%	-0.88	0.377
If support in the home language (e.g., Spanish) is not continued during the school years, dual language learning children will not become fluent bilinguals.	2.72	0.77	6.3%	28.1%	53.1%	12.5%	65.6%	1.59	0.112
When children mix two languages in their communication (e.g., mixing words from two languages in a sentence), they are confusing the two languages as being one.	2.25	0.62	9.4%	56.3%	34.4%	0.0%	65.7%	1.59	0.112
Young dual language learners are often confused about which language to speak in social situations.	2.09	0.64	15.6%	59.4%	25.0%	0.0%	75.0%	2.65	0.008
Immigrant parents whose children are showing language delays should stop using the home language and speak only English so as not to confuse the children.	2.00	0.68	22.6%	54.8%	22.6%	0.0%	77.4%	2.87	0.004
Children who are exposed to two languages may experience delays in cognitive development due to the dual-language exposure.	1.74	0.86	48.4%	32.3%	16.1%	3.2%	80.7%	3.23	0.001
Hearing two or more languages in childhood may cause confusion and put children at greater risk for language delay or impairment.	1.94	0.67	25.0%	56.3%	18.8%	0.0%	81.3%	3.36	0.001
Use of the home language (e.g., Spanish) by children or parents at home slows down children’s English learning and should be discouraged.	1.69	0.69	43.8%	43.8%	12.5%	0.0%	87.6%	4.07	0.000
Although dual language learners may show delays in their second language initially, they can catch up to monolingual children (i.e., children who only speak English) if they receive enough support.	3.13	0.56	0.0%	9.7%	67.7%	22.6%	90.3%	4.31	0.000
If rich dual language input is maintained, dual language learners can catch up with their monolingual peers.	3.03	0.54	3.1%	3.1%	81.3%	12.5%	93.8%	4.77	0.000
Preschool programs that support home language (e.g., Spanish) benefit children’s development of English skills.	3.14	0.44	0.0%	3.4%	79.3%	17.2%	96.5%	4.83	0.000

#### Children’s Home Dual Language Experiences

Parents reported on the language(s) children heard from mother, father, younger sibling(s), older sibling(s), other adult(s) in the household, and children’s friends respectively (i.e., *language input*; 1-Only Spanish, 5-Only English), and children’s use of English and Spanish with these social partners respectively on the same scale (i.e., *language output*; see Branum-Martin et al., 2014). An average score was calculated for dual language input and output, respectively.

Additionally, parents reported on how frequently family members engaged in language and literacy activities in English and Spanish with their children (i.e., reading, telling stories, teaching letters, rhyming games, and teaching words; 1-Never, 5-Everyday; Lonigan and Farver, unpublished). An average score was calculated to indicate the *frequency of language and literacy activities* in English and Spanish, respectively.

#### Children’s School Readiness Skills

Both the lead teacher and teaching assistant in each classroom rated children’s school readiness skills in English on 12 items (e.g., “Can express his/her needs, wants, and thoughts in age-appropriate English.”; 1-not yet, 5-proficient; adapted from the Early Childhood Longitudinal Study, Kindergarten; National Center for Educational Statistics, 1998). Children’s school readiness skills in Spanish were rated by the lead teacher and/or teaching assistant who understood Spanish using the same items. Ratings from the lead teachers and teaching assistants were highly correlated (English: *r* = 0.91, *p* < 0.001; Spanish: *r* = 0.74, *p* < 0.001). A composite score was created for each language by averaging both teachers’ ratings on all the items.

### Missing Values

Two percent (4 out of 192 responses) of the demographic variables were missing and were imputed by SPSS’s multiple imputation. We presented below the pooled results from 20 imputed datasets. There were no missing data for parental beliefs and knowledge and children’s home dual language experiences. The teacher-rated school readiness data were missing for 5 children, because both the teacher and teaching assistant in the classroom were on sick leave during data collection, and the new teachers did not know the children well enough to provide the ratings. These children were excluded from the analyses involving school readiness outcomes.

## Results

Descriptive statistics for child and parent demographic variables, parental dual language beliefs and knowledge, children’s home dual language experiences, and children’s teacher-rated school readiness skills are presented in Tables 1, 2. The families had relatively low income, with 83.4% having an annual household income of no more than $50,000, way below the local region’s median household income for a family of four at $74,533 (The American Community Survey, 2019) and the national median household income of $68,703 in 2019 (Semega et al., 2020).

**TABLE 2:** Descriptive statistics for demographic variables, children’s home dual language experiences, and children’s school readiness skills.

	Mean (*SD*) or%	Min	Max
**Child characteristics**			
Age (months)	48.88 (5.40)	37.82	65.15
Males	37.50%		
**Parent characteristics**			
Age (years)	32.47(8.33)	22	69
**Ethnicity**			
Latino	75%		
African American	25%		
**Educational level**			
1-Lower than high school	9.4%		
2-High school or GED	37.5%		
3-Some college	15.6%		
4-Associate degree	12.5%		
5-Bachelor’s degree	21.9%		
6-Graduate degree	3.1%		
**Household annual income^a^**			
1-<$5k	10.0%		
2-$5k-$15k	13.3%		
3-$15k-$25k	26.7%		
4-$25k-$50k	33.3%		
5- $50k-$75k	10.0%		
6-$75k-$100k	3.3%		
7->$100k	3.3%		
Native born	60%		
Years living in the United States (for foreign born only)	20.75(9.31)	10	38
English proficiency^b^	3.38(1.05)	1	4
Spanish proficiency^b^	2.83 (1.20)	1	4
**Home dual language experiences**			
Language input^c^	3.66(0.88)	1.8	4.83
Language output^d^	4.01(1.06)	1.4	5
English language and literacy activities^e^	3.89(0.92)	1.33	5
Spanish language and literacy activities^e^	2.54(1.19)	1	4.67
Children’s school readiness skills in English^f^	3.13(0.96)	1.67	4.83
Children’s school readiness skills in Spanish^f^	1.80(1.03)	1	4.63

*^*a*^At the time of data collection, the region’s median household income for a family of four was $74,533 (The American Community Survey, 2019) and the national median household income for a family of four was $68,703 in 2019 (Semega et al., 2020).*

*^*b*^Average score of 4 items examining parents’ ability to understand, speak, read, and write in English and Spanish (1-Not well at all, 2-Not well, 3- Well, 4- Very well).*

*^*c*^Average score of 6 items on the types of language mother, father, older siblings, younger siblings, other adults, and friends use when speaking to the target child (1-Spanish only, 2-Mostly Spanish, 3-Spanish and English equally, 4-Mostly English, 5-English Only).*

*^*d*^Average score of 6 items on the types of language the target child use when speaking to mother, father, older siblings, younger siblings, other adults, and friends (1-Spanish only, 2-Mostly Spanish, 3-Spanish and English equally, 4-Mostly English, 5-English Only).*

*^*e*^Average frequency of family members engaging in different language and literacy activities (i.e., reading, telling stories, teaching letters, rhyming games, and teaching words) with the target child (1-Never, 2-Less than once a month, 3-A few times a month, 4-A few times a week, 5-Everyday).*

*^*f*^Teachers’ average rating of children’s school readiness skills in English and Spanish (12 items for each language; 1-not yet, 2-beginning, 3-in progress, 4-intermediate, 5-proficient).*

### Parents’ Beliefs and Knowledge About Dual Language Development and Practices

As a group, parents scored 2.87 (*SD* = 0.30, range = 2.33–4.00) on the parental beliefs and knowledge survey and had an average correct rate of 71% (*SD* = 0.15; range = 33%–100%), suggesting that in general, parental dual language beliefs and knowledge were more consistent with scientific evidence than not. At the same time, there existed substantial variation in parents’ beliefs and knowledge. One parent responded to all statements correctly, whereas some parents responded to less than half of the statements correctly.

Table 1 presents descriptive statistics for parental responses. Among the 15 statements, the correct rates ranged from 31.3% to 96.5%. Non-parametric binomial tests suggested that the correct rates of 6 statements were significantly greater than chance at the Bonferroni-adjusted significance level (*p* < 0.05/15 = 0.003; see Table 1).

Most parents believed that dual language exposure does not increase the risk for cognitive or language delays (81%), and that immigrant parents whose children have language delays should continue using their home language (77%). Similarly, 75% of parents believed that young DLLs understand which language to speak in social situations. However, 44% of parents viewed code-switching (e.g., mixing words from two languages in a sentence) as a sign of confusion, even though research has shown that it is a natural characteristic of dual language development (Hammer et al., 2014).

When comparing dual language and monolingual development, over 90% of parents believed that DLLs are able to catch up with their monolingual peers in English when receiving sufficient support. Yet, parents were less certain about whether DLL children’s total growth in both languages (e.g., total vocabulary of English and Spanish) is comparable to monolingual children’s skills in one language, with only 57% of parents agreeing with this statement.

In terms of practices to support dual language development, although most parents (88%) believed that the use of home language by children or parents at home does not slow down English development, some parents still had misconceptions and misunderstanding. For example, 69% of our sample agreed that immigrant parents should speak English as much as possible with their children to support their children’s school learning; about 50% thought that parents can only support a child’s dual language development if they can speak English and another language; and about 34% did not realize that continued support in the home language during the school years is necessary for DLLs to become fluent bilinguals.

Finally, parents held mixed beliefs about dual language education. Almost all parents (97%) believed that preschool programs that support home language benefit children’s English development. Yet, about 40% of the parents considered English-only programs as the best way to support DLLs’ English learning, and about 48% of parents agreed that dual language programs could be harmful to English-speaking children.

### Relating Parental Beliefs and Knowledge to Demographic Factors

We conducted correlations and *t*-tests to examine whether parental beliefs and knowledge varied by their SES, immigration, and linguistic backgrounds. Parental education (*r* = 0.37, *p* = 0.036) and household income (*r* = 0.51, *p* = 0.007) were positively correlated with parental beliefs and knowledge. Parental beliefs and knowledge did not vary by their native-born status (*t*(30) = 0.12, *p* = 0.907), English proficiency (*r* = 0.20, *p* = 0.276), or Spanish proficiency (*r* = 0.15, *p* = 0.408).

### Relating Parental Beliefs and Knowledge to Children’s Home Dual Language Experiences and School Readiness Skills

We first conducted bivariate correlations to examine whether parental beliefs and knowledge were associated with children’s dual language experiences at home and their school readiness skills in English and Spanish. Parental beliefs and knowledge were not significantly correlated with dual language input (*r* = −0.13, *p* = 0.483) and output (*r* = −0.01, *p* = 0.969), the frequencies of English (*r* = −0.19, *p* = 0.301) and Spanish (*r* = −0.10, *p* = 0.601) language and literacy activities, or children’s school readiness skills in English (*r* = 0.21, *p* = 0.289) and Spanish (*r* = 0.29; *p* = 0.147). These bivariate correlations were not significant, probably because they did not account for the variations in parents’ English and Spanish proficiency and other demographic factors. It is therefore important to examine how parental beliefs and knowledge relate to children’s dual language experiences and school readiness outcomes in multiple regression models that control for relevant individual and contextual factors.

We next conducted multiple regression models, using parental beliefs and knowledge as the key predictor, and the dual language input and output, the frequencies of language and literacy activities in English and Spanish at home, and children’s school readiness skills in English and Spanish as the dependent variables. We examined whether each dependent variable was associated with any of the demographic variables (i.e., parental education, ethnicity, native-born status, and language proficiency as well as household income and child age). Demographic variables that were correlated with each dependent variable were controlled in the respective regression models.

As shown in Table 3, parental beliefs and knowledge were associated with relatively more Spanish input at home (*b*(*SE*) = −0.62(0.28), *beta* = −0.21, *p* = 0.028; *F*(6, 25) = 18.92, R^2^ = 0.82, *p* < 0.001; see Model 1) but were not related to children’s dual language output (*p* = 0.287; see Model 2), after controlling for parents’ English and Spanish proficiency, native born status, educational level, and ethnicity. Furthermore, parental beliefs and knowledge were negatively associated with the frequency of English language and literacy activities (*b*(*SE*) = −0.88(0.38), *beta* = −0.29, *p* = 0.019; *F*(5, 26) = 9.98, *R^2^* = 0.66, *p* < 0.001; see Model 3), after controlling for parents’ English and Spanish proficiency, native born status, and child age. However, parental beliefs and knowledge did not significantly predict the frequency of Spanish activities (*p* = 0.183; see Model 4), above and beyond parents’ English and Spanish proficiency, native born status, and ethnicity. Finally, parental beliefs and knowledge were positively associated with children’s school readiness skills in Spanish (*b*(*SE*) = 1.91(0.50), *beta* = 0.35, *p* = 0.016; *F*(4, 22) = 8.72, *R^2^* = 0.61, *p* = 0.016; see Model 6), after controlling for parents’ English and Spanish proficiency and native born status. The prediction was not significant for school readiness skills in English (*p* = 0.211; see Model 5) after controlling for child age. Given the small sample size and the number of predictors, our models are under-powered and prone to error, so regression findings need to be interpreted with caution.

**TABLE 3:** Parental beliefs and knowledge predicting children’s home dual language experiences and school readiness skills.

	Model 1: Dual language input^a^					Model 2: Dual language output^a^				
		
	*B*	*SE*	*Beta*	*t*	*p*	*B*	*SE*	*Beta*	*t*	*p*
Parent English proficiency	0.38	0.11	0.45	3.59	0.000	0.70	0.17	0.69	4.04	0.000
Parent Spanish proficiency	−0.38	0.10	−0.51	−3.63	0.000	−0.36	0.18	−0.40	−2.03	0.042
Parent native born	−0.11	0.25	−0.06	−0.43	0.670	−0.74	0.44	−0.35	−1.68	0.094
Parent education	0.13	0.07	0.21	1.86	0.063	0.14	0.12	0.19	1.17	0.241
Latino	−0.14	0.25	−0.07	−0.56	0.579	−0.15	0.41	−0.06	−0.36	0.722
Parental beliefs and knowledge	−0.62	0.28	−0.21	−2.20	0.028	−0.50	0.47	−0.14	−1.06	0.287
Model fitness	*F*(6, 25) = 18.92, *R^2^* = 0.82, *p* < 0.001					*F*(6, 25) = 8.60, *R*^2^ = 0.67, *p* < 0.001				

	**Model 3: English language and literacy activities**					**Model 4: Spanish language and literacy activities**				
		
	***B***	***SE***	***Beta***	***t***	***p***	***B***	***SE***	***Beta***	***t***	***p***

Parent English proficiency	0.48	0.15	0.55	3.14	0.002	−0.02	0.21	−0.02	−0.09	0.929
Parent Spanish proficiency	−0.04	0.11	−0.05	−0.36	0.721	0.58	0.20	0.58	2.86	0.004
Parent native born	0.25	0.33	0.14	0.75	0.454	−0.52	0.48	−0.22	−1.08	0.282
Latino	−^*b*^	−	−	−	−	0.08	0.49	0.03	0.16	0.874
Child age	−0.06	0.02	−0.32	−2.68	0.007	−	−	−	−	−
Parental beliefs and knowledge	−0.88	0.38	−0.29	−2.34	0.019	−0.72	0.54	−0.18	−1.33	0.183
Model fitness	*F*(5, 26) = 9.98, *R*^2^ = 0.66, *p* < 0.001					*F*(5, 26) = 6.99, *R*^2^ = 0.57, *p* < 0.001				

	**Model 5: Teacher−rated school readiness in English**					**Model 6: Teacher−rated school readiness in Spanish**				
		
	***B***	***SE***	***Beta***	***t***	***p***	***B***	***SE***	***Beta***	***t***	***p***

Parent English proficiency	−	−	−	−	−	−0.41	0.19	−0.42	−2.19	0.029
Parent Spanish proficiency	−	−	−	−	−	−0.03	0.15	−0.03	−0.19	0.850
Parent native born	−	−	−	−	−	−0.85	0.40	−0.41	−2.14	−0.033
Child age	0.08	0.03	0.41	2.27	0.023	−	−	−	−	−
Parental beliefs and knowledge	0.71	0.57	0.22	1.25	0.211	1.19	0.50	0.35	2.40	0.016
Model fitness	*F*(2, 24) = 3.28, *R*^2^ = 0.22, *p* = 0.055					*F*(4, 22) = 8.72, *R*^2^ = 0.61, *p* = 0.016				

*^*a*^Higher value indicates relatively more English and less Spanish input/output at home.*

*^*b*^Demographic variables that were not associated with the dependent variable at the bivariate level were not included in the model.*

## Discussion

In this study we examined the beliefs and knowledge parents of Spanish-English DLLs had about dual language development and practices to support DLLs. We also asked how parental beliefs and knowledge were associated with their socioeconomic and sociocultural backgrounds, as well as children’s dual language experiences at home and school readiness skills. Three key findings emerged. First, parents showed both strengths and misconceptions in their understanding of dual language development and practices. Second, parents from higher-SES backgrounds had dual language beliefs and knowledge that were more in line with scientific evidence. Third, parental beliefs and knowledge were associated with some, but not all, aspects of children’s dual language experiences, as well as children’s school readiness skills in Spanish. Due to the small sample size, our investigation was exploratory, and the findings should be interpreted cautiously. Nonetheless, the current study has both theoretical and practical contributions. Theoretically, the current study expanded prior evidence of the role of parent-child relationship and interactions on children’s social and cognitive development, such as individual’s behavior (Berscheid, 1999; Reis et al., 2000), prosociality (Eisenberg et al., 2016), and evaluation processes (Geraci, 2020; Marshall et al., 2020), by applying the sociocultural theory, the bioecological model, and the developmental niche theory to the dual language learning context. Practically, the study added to the limited quantitative empirical data regarding parental beliefs and knowledge about dual language development and its relations with DLLs’ dual language experiences and developmental outcomes, thereby informing the development of caregiver-focused language interventions for DLL families. We discuss the three key findings and their theoretical and practical imports in each of the following subsections.

### Strengths and Misconceptions in Parental Beliefs and Knowledge

Parents in our sample displayed multiple strengths in their dual language beliefs and knowledge. Most parents recognized the benefits of supporting home language development in the home and school contexts and rejected the false beliefs that dual language exposure causes or worsens cognitive or language delays. These beliefs are consistent with prior findings that bilingualism and home language growth benefit children’s social and cognitive development, such as academic performance (Tamis-LeMonda et al., 2014b), socioemotional adjustment (Hammer et al., 2014), family relationship (Oh and Fuligni, 2010), metalinguistic awareness (Diaz and Farrar, 2018), and theory of mind (Siegal et al., 2010). These evidence-based beliefs should be recognized as strengths and reinforced in parent education and intervention programs to motivate and empower parents of DLLs.

Nonetheless, there existed opportunities for improvement. Parents tended to misperceive characteristics of dual language development as indicators of problem or disadvantage. Many of them viewed code-switching as a sign of confusion and did not realize that when there is sufficient support in either language, DLLs’ total growth across the two languages is comparable to monolingual children’s skills in one language. Yet, empirical research has suggested that code-switching is a normal and natural phenomenon in the course of dual language development (Hammer et al., 2014), and that DLLs’ total language knowledge across the two languages is similar to, if not larger than, that of monolingual children (Pearson et al., 1993; Thordardottir et al., 2006; Hoff et al., 2012). These misconceptions of parents could lead to unnecessary concerns about children’s language development and discourage parents from exposing children to two languages (Lee et al., 2015; Cycyk and Hammer, 2020). Additionally, some parents underestimated the importance of continued support in the home language and viewed using English at home and school as the best way to promote DLLs’ English and academic growth. Ample evidence has suggested that dual language programs are equally, if not more, effective in supporting English development compared to English-only programs (Barnett et al., 2007; Collier and Thomas, 2017), and that the quality of language input is more important than which language children hear (Paradis, 2011; Song et al., 2012, 2021). However, this set of knowledge still needs to be communicated to parents of DLLs. Together, these findings highlight specific areas of parental dual language beliefs and knowledge that need to be improved, thereby informing the development of culturally responsive interventions and services.

### Parental Beliefs and Knowledge in Relation to Demographic Factors

We found enormous variations in parental dual language beliefs and knowledge (see also Mancilla-Martinez and Lesaux, 2014; Hwang et al., 2020). More importantly, these variations were associated with SES. Parents with higher levels of education and income showed greater levels of dual language beliefs and knowledge. This finding is consistent with prior work on SES differences in parents’ knowledge about general infant development and early language and literacy development (Davis et al., 2016; Rowe et al., 2016; Gonzalez et al., 2017; Suskind et al., 2018). There is evidence that parents gain dual language beliefs and knowledge from professionals and scientific research (King and Fogle, 2006). Parents from higher-SES background might have greater access to these sources of information (Rowe et al., 2016). Therefore, there is a need to make research-based information on dual language development and practices more accessible to low-SES parents in order to enhance their knowledge.

We did not find any association between parental beliefs and knowledge and their native-born status or English or Spanish proficiencies. These findings challenge the deficit view of the knowledge and practices of immigrant parents (Song, 2019). While being native-born and being proficient in English may be related to higher levels of parental knowledge in other areas of child development (e.g., general development and early language development; Glick et al., 2009; Keels, 2009; Suskind et al., 2018) through more exposure to such knowledge, immigrant parents’ own dual language experiences might provide unique knowledge and skills, as part of the households’ Funds of Knowledge, to support DLLs’ development and learning at school (González, 2005; King and Fogle, 2006).

### Parental Beliefs and Knowledge in Relation to Children’s Home Dual Language Experiences and School Readiness Skills

In line with the sociocultural perspectives, which emphasize the critical role of parents and parent-child interactions in children’s language acquisition (Vygotsky, 1978), our findings revealed that parental beliefs and knowledge might shape how they construct children’s dual language learning environment and impact DLLs’ development outcome. Higher levels of parental beliefs and knowledge predicted more Spanish input at home and less frequent English language and literacy activities. Parents who had views that were more consistent with research evidence might recognize the importance of providing sufficient and sustained home language exposure to dual language development, therefore placing more emphasis on Spanish input at home but considering school to be more responsible to teach children English (Adair and Tobin, 2008; Lee et al., 2015). However, parental beliefs and knowledge were not related to children’s dual language output or the frequency of Spanish activities. Perhaps children’s dual language output is influenced by their own language preference or language skills rather than their parents’ expectations. Additionally, parents may not be aware of the importance of engaging DLLs in language and literacy activities in Spanish beyond merely speaking Spanish with them or have limited time and resources for these activities (Liang, 2018).

Parental beliefs and knowledge predicted children’s school readiness skills in Spanish but not in English. These findings offer preliminary support for the link between parental beliefs and knowledge and DLLs’ school readiness skills. Although the current study did not have enough power to test mediation models, parents with greater knowledge of dual language development and practices may better support DLLs to develop school readiness through providing Spanish input at home. Spanish input may strengthen parent-child relationship (Oh and Fuligni, 2010; Liang, 2018; Cycyk and Hammer, 2020), which further supports language and cognitive development. For instance, there is evidence that DLL children’s Spanish fluency was related to the quality of parent-child relationship, which in turn predicted children’s school performance (Schofield et al., 2012). It is also possible that parents with children who showed stronger school readiness skills in Spanish developed stronger beliefs about children’s ability of learning two languages and the benefits of bilingualism.

### Limitations

The study has several limitations. First, given the exploratory nature of the study, the small sample size, and the relatively large age range of the target children (3- to 5-year-old), findings must be interpreted with caution and replicated in future work. In particular, more work is needed to understand whether these findings are generalizable to DLL children in varying age groups and families from different linguistic, cultural, and socioeconomic backgrounds. It would also be meaningful to further ask whether the association between parental beliefs and knowledge and child outcome is mediated by children’s dual language experiences at home. Additionally, our survey of parental dual language beliefs and knowledge is still preliminary. We are currently revising the survey to capture a more comprehensive picture of parental dual language beliefs and knowledge (e.g., how to provide high-quality language environment for DLLs, DLLs’ developmental milestones), which will be given to a larger and more diverse sample of parents of DLLs, allowing assessment of the psychometric property of the instrument. Furthermore, the study was correlational, which precludes causal inferences. Experimental and longitudinal work is needed to examine how parental beliefs and knowledge is related causally to DLLs’ dual language experiences and developmental outcomes and how parental beliefs and knowledge might change over time. Finally, we used teacher reports of children’s school readiness skills. Future research should also include direct and/or observational assessments and examine other developmental outcomes.

## Conclusion

Understanding parental dual language beliefs and knowledge is a necessary step to supporting families of DLLs. This study revealed both accuracy and misconceptions in parental dual language beliefs and knowledge. Parental dual language beliefs and knowledge varied by SES and were associated with DLLs’ dual language experiences at home and school readiness skills in Spanish. Together, these findings highlight the essential role parents play in dual language development. There is a need for culturally responsive interventions and parent education programs, which must recognize both the strengths and areas of improvement in parents of DLLs and support parents to transform knowledge into high-quality language and literacy experiences that benefit DLLs.

## Data Availability Statement

The datasets presented in this article are not readily available because only members of the research team are allowed to have access to the data. Requests to access the datasets should be directed to rufan.luo@rutgers.edu.

## Ethics Statement

The study was reviewed and approved by the Rutgers University Institutional Review Board. Written informed consent to participate in this study was provided by the participants or the participants’ legal guardian.

## Author Contributions

RL contributed to the study design, data collection, data analysis, and manuscript writing. LS contributed to the study design, data analysis, and manuscript writing. CV and GS-B contributed to the data collection. All authors contributed to the article and approved the submitted version.

## Conflict of Interest

The authors declare that the research was conducted in the absence of any commercial or financial relationships that could be construed as a potential conflict of interest.
